# Supplementary material for: Distinct interacting cortical networks for stimulus-response and repetition-suppression
Source: Commun Biol. 2022 Sep 5;5:909. doi: 10.1038/s42003-022-03861-4 (PMC9445181; doi:10.1038/s42003-022-03861-4)
Supplement: Supplementary file 2 — reporting summary [file 42003_2022_3861_MOESM2_ESM.pdf]

nature portfolio

Corresponding author(s): Stefan Dürschmid, stefan.duerschmid@lin-magdeburg.de

Last updated by author(s): Jul 17, 2022

Reporting Summary

Nature Portfolio wishes to improve the reproducibility of the work that we publish. This form provides structure for consistency and transparency in reporting. For further information on Nature Portfolio policies, see our Editorial Policies and the Editorial Policy Checklist.

Statistics

For all statistical analyses, confirm that the following items are present in the figure legend, table legend, main text, or Methods section.

n/a	Confirmed	
☐	☒	The exact sample size (*n*) for each experimental group/condition, given as a discrete number and unit of measurement
☐	☒	A statement on whether measurements were taken from distinct samples or whether the same sample was measured repeatedly
☐	☒	The statistical test(s) used AND whether they are one- or two-sided<br>*Only common tests should be described solely by name; describe more complex techniques in the Methods section.*
☐	☒	A description of all covariates tested
☐	☒	A description of any assumptions or corrections, such as tests of normality and adjustment for multiple comparisons
☐	☒	A full description of the statistical parameters including central tendency (e.g. means) or other basic estimates (e.g. regression coefficient) AND variation (e.g. standard deviation) or associated estimates of uncertainty (e.g. confidence intervals)
☐	☒	For null hypothesis testing, the test statistic (e.g. F, t, r) with confidence intervals, effect sizes, degrees of freedom and P value noted<br>*Give P values as exact values whenever suitable.*
☐	☒	For Bayesian analysis, information on the choice of priors and Markov chain Monte Carlo settings
☐	☒	For hierarchical and complex designs, identification of the appropriate level for tests and full reporting of outcomes
☐	☒	Estimates of effect sizes (e.g. Cohen's *d*, Pearson's *r*), indicating how they were calculated

Our web collection on statistics for biologists contains articles on many of the points above.

Software and code

Policy information about availability of computer code

Data collection	The stimuli were generated using Cool Edit 2000 software (Syntrillium) on computers running on Windows XP. ECoG was recorded at UCSF using electrode grids equipped with 64 platinum-iridium-electrodes, arranged in an 8 x 8 array with 10 mm center-to-center spacing (Ad-Tech Medical Instrument Corporation, Racine, Wisconsin). At The Mara, Bielefeld, ECoG was recorded via electrode strips (single strips or parallel arrangement of strips; white dots in Fig 1b,d represent all electrode locations) using a Nihon Kohden amplifier (Tokyo, Japan). Electrodes were positioned based solely on clinical needs. Exposed electrode diameter was 2.3 mm. The data at UCSF were recorded continuously throughout the task at a sampling rate of 2003 Hz. At The Mara, sampling rate was 2000 Hz in the case of four subjects and 1000 Hz in one subject.
Data analysis	We used Matlab 2013b (Mathworks, Natick, USA) for all offline data processing. All filtering was done with zero-shift infinite impulse response (IIR) filters [Butterworth filter of order 4: filtfilt() function in matlab].

For manuscripts utilizing custom algorithms or software that are central to the research but not yet described in published literature, software must be made available to editors and reviewers. We strongly encourage code deposition in a community repository (e.g. GitHub). See the Nature Portfolio guidelines for submitting code & software for further information.

Data

Policy information about availability of data

All manuscripts must include a data availability statement. This statement should provide the following information, where applicable:

- Accession codes, unique identifiers, or web links for publicly available datasets
- A description of any restrictions on data availability
- For clinical datasets or third party data, please ensure that the statement adheres to our policy

The datasets generated and/or analyzed during the current study are available in the Open Science Foundation repository (https://osf.io/ceutw/?view_only=1d6ba767ebe3458ca26ade4945972d8c).

Human research participants

Policy information about studies involving human research participants and Sex and Gender in Research.

Reporting on sex and gender	Sex was determined based on clinical information on patients. We did not assign gender or analyze gende as the purpose was not to gain sex- or gender-related results in our study.
Population characteristics	The participants were epilepsy patients that met the inclusion criteria (pharmako-resistant epilepsy, no macroscopical structural brain damage, adult, consent to epilepsy surgery).
Recruitment	Subjects were asked for participation when subjected to presurgical monitoring for drug-resistant epilepsy at one of the two epilepsy centres at the University of California in San Fransisco (UCSF) and the Dept of Epileptology at Krankenhaus Mara, Bielefeld University.
Ethics oversight	Recordings were approved by the local ethic committees: Commitee for the Protection of Human Subjects at University of California in Berkeley and Ethics Committee of the Ott-von-Guericke-University Magdeburg

Note that full information on the approval of the study protocol must also be provided in the manuscript.

Field-specific reporting

Please select the one below that is the best fit for your research. If you are not sure, read the appropriate sections before making your selection.

☒ Life sciences ☐ Behavioural & social sciences ☐ Ecological, evolutionary & environmental sciences

For a reference copy of the document with all sections, see nature.com/documents/nr-reporting-summary-flat.pdf

Life sciences study design

All studies must disclose on these points even when the disclosure is negative.

Sample size	The sample was recruited according to inclusion criteria (pharmako-resistant epilepsy, no macroscopical structural brain damage, adult, consent to epilepsy surgery). We reviewed a priori previous ECoG studies on predictive coding mechanisms as to the number of subjects and found that the majority used around 7 (SD 4) subjects.<br>We sought to include 10 participants to exceed the standard sample size in ECoG experiments. We designed the experiment to collect large numbers of trials in each participant and each block for rare conditions (per block 400 trials including 320 standard trials) in order to provide large statistical power for an ECoG study even after allowing for artifact rejection.
Data exclusions	After visual inspection, we excluded channels exhibiting ictal activity or excessive noise from further analysis. In the remaining "good" channels we then excluded time intervals containing artifactual signal distortions such as signal steps or pulses by visual inspection.
Replication	We used auditory stimuli that have been used before and have been reported in numerous studies. Stimuli and procedure are common for ieeg experiments and therefore results should be well reproducable.
Randomization	Allocation of conditions was randomized blockwise (400 trials per block) within each participant.
Blinding	Due to the non-clinical or related purpose of the study (i.e., no comparison between causes of conscious/behavioral effects) , blinding was not relevant.

Reporting for specific materials, systems and methods

We require information from authors about some types of materials, experimental systems and methods used in many studies. Here, indicate whether each material, system or method listed is relevant to your study. If you are not sure if a list item applies to your research, read the appropriate section before selecting a response.

Materials & experimental systems

n/a	Involved in the study
☒	☐ Antibodies
☒	☐ Eukaryotic cell lines
☒	☐ Palaeontology and archaeology
☒	☐ Animals and other organisms
☒	☐ Clinical data
☒	☐ Dual use research of concern

Methods

n/a	Involved in the study
☒	☐ ChIP-seq
☒	☐ Flow cytometry
☒	☐ MRI-based neuroimaging
